# High‐Performance Non‐Fullerene Organic Solar Cells Based on a Selenium‐Containing Polymer Donor and a Twisted Perylene Bisimide Acceptor

**DOI:** 10.1002/advs.201600117

**Published:** 2016-04-23

**Authors:** Tao Liu, Dong Meng, Yunhao Cai, Xiaobo Sun, Yan Li, Lijun Huo, Feng Liu, Zhaohui Wang, Thomas P. Russell, Yanming Sun

**Affiliations:** ^1^ Heeger Beijing Research and Development CenterSchool of Chemistry and EnvironmentBeihang UniversityBeijing100191P. R. China; ^2^ Beijing National Laboratory for Molecular ScienceKey Laboratory of Organic SolidsInstitute of ChemistryChinese Academy of SciencesBeijing100190P. R. China; ^3^ Materials Science DivisionLawrence Berkeley National LabBerkeleyCA94720USA; ^4^ Polymer Science and Engineering DepartmentUniversity of MassachusettsAmherstMA01003USA

**Keywords:** organic solar cells, perylene bisimide acceptor, polymer donor, power conversion efficiency

## Abstract

**A novel polymer donor (PBDTS‐Se)** is designed to match with a non‐fullerene acceptor (SdiPBI‐S). The corresponding solar cells show a high efficiency of 8.22%, which result from synergetic improvements of light harvesting, charge carrier transport and collection, and morphology. The results indicate that rational design of novel donor materials is important for non‐fullerene organic solar cells.

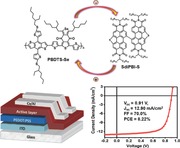

Non‐fullerene acceptors have attracted tremendous interest due to their potential use as alternatives to the ubiquitous fullerene derivatives in bulk heterojunction (BHJ) organic solar cells.^1^, ^2^, ^3^, ^4^, ^5^, ^6^ Extensive studies have been carried out to develop polymeric and small molecule acceptors over the past decade.^7^, ^8^, ^9^, ^10^, ^11^, ^12^, ^13^, ^14^ A number of high performance systems have been recently reported with power conversion efficiencies (PCEs) exceeding 8%, comparable to and exceeding BHJ organic solar cells made from fullerene acceptors in performance.^15^, ^16^, ^17^, ^18^

The inefficacy of tuning frontier energy levels of fullerenes constrains donor material development.^19^, ^20^, ^21^, ^22^ Currently, the donor material design must reference the lowest unoccupied molecular orbital (LUMO) level of fullerene derivatives (e.g., [6,6]‐phenyl C_71_‐butyric acid methyl ester, PC_70_BM) to optimize open‐circuit voltage (*V*_oc_).^23^ Other disadvantages, such as the weak absorption in the visible region, high production cost, and poor photochemical stability, make fullerenes less than an ideal acceptor material for BHJ organic solar cells. In contrast, non‐fullerene acceptors are much more versatile in chemistry. The optical properties and the energy levels can be fine‐tuned by structural modification. And thus there are more opportunities to group donor/acceptor pairs, to form better frontier energy level offsets and to complement light absorption.^24^, ^25^ Thus, short‐circuit current (*J*_sc_) and *V*_oc_ can be optimized to achieve a better power conversion efficiency.

Many non‐fullerene acceptors have been developed. At the beginning, poly(3‐hexylthiophene) (P3HT) was selected as the donor material for the fabrication of non‐fullerene solar cells. However, due to its narrow absorption and relatively high‐lying occupied molecular orbital (HOMO) energy level, P3HT‐based non‐fullerene solar cells suffer from low PCEs, mainly because of the low *J*_sc_ and fill factor (FF) values.^26^ Later, donor–acceptor (D–A) alternating copolymers with better absorption were introduced, which showed improved device efficiencies.^27^ Choosing a suitable donor material to pair with nonfullerence acceptor is critical. Both the energy level alignment and blend morphology need to be synergistically optimized. One successful example was shown by Jenekhe and co‐workers, who reported on a 3,4‐ethylenedioxythiophene‐linked arylene diimide acceptor (DBFI‐EDOT). The choice of suitable BDT‐based copolymer (PBDTT‐FTTE) and thiazolothiazole‐dithienosilole copolymer (PSEHTT) that paired with DBFI‐EDOT yielded a PCE of 8.5% with a high *J*_sc_ (15.67 mA cm^−2^) and a high *V*_oc_ (0.91 V) showing the importance of donor materials selection.^17^ In this contribution, we designed and synthesized a novel polymer donor, where a benzodithiophene (BDT) derivative with large π‐conjugated side chain is used as the electron‐rich donor subunit and 1,3‐di(thiophen‐2‐yl)‐selenopheno[3′,4′:4,5]benzo[1,2‐c]thiophene‐4,8‐dione is used as the electron‐deficient acceptor subunit (PBDTS‐Se). The incorporation of a selenium heteroatom in the conjugated polymers results in a higher photovoltaic performance than its sulfur counterpart, since selenium has a larger and looser electron cloud than sulfur, which improves the intramolecular Se–Se interaction and facilitates carrier transport.^28^, ^29^ This new donor polymer is paired with our recently developed bay‐linked sulfur‐containing perylene bisimide (PBI) dimer (SdiPBI‐S). Previous studies using SdiPBI‐S acceptor yielded a PCE of 7.16%.^16^ The better optimized donor polymer matches well with SdiPBI‐S, resulting in improvements in device parameters. A PCE of 8.22%, with a *J*_sc_ of 12.90 mA cm^−2^, *V*_oc_ of 0.91 V, and FF of 70.0% was found. The improved performance resulted from synergetic improvement of light harvesting, charge carrier transport and collection, and morphology.

The chemical structures of PBDTS‐Se and SdiPBI‐S are shown in **Figure** **1**a. The detailed synthesis of PBDTS‐Se is shown in **Scheme** **1**. Electronic‐rich dialkylthio‐substituted BDT monomer is prepared according to the literature procedures.^30^, ^31^ Dibranched 2‐ethylhexyl were linked to selenophene in one step, then a typical acylation reaction was carried out between dialkyl substituted selenophene and 2,5‐dibromothiophene‐3,4‐dicarbonyl dichloride to get a key intermediate (4). PBDTS‐Se was prepared through the Stille coupling reaction between the bis(trimethyltin) monomers and Se. PBDTS‐Se exhibits good solubility in commonly used solvents, such as chloroform (CHCl_3_) and *o*‐dichlorobenzene (*o*‐DCB) at room temperature and thermally stable up to temperatures beyond 287 °C under an inert atmosphere (Figure S1, Supporting Information).

**Figure 1:**
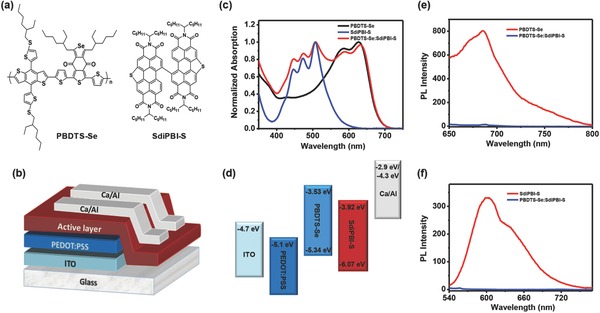
a) Chemical structures of PBDTS‐Se:SdiPBI‐S. b) The configuration of conventional device structure used in this study. c) Normalized UV–vis absorption spectra of PBDTS‐Se, PBDTS‐Se, and PBDTS‐Se:SdiPBI‐S films. d) Energy levels of diagrams of all materials used in this study. e) The photo­luminescent properties of PBDTS‐Se, PBDTS‐Se:SdiPBI‐S (1:1, w/w) films (excitation at 627 nm). f) The photoluminescent properties of SdiPBI‐S, PBDTS‐Se:SdiPBI‐S (1:1, w/w) films (excitation at 507 nm).

**Scheme 1:**
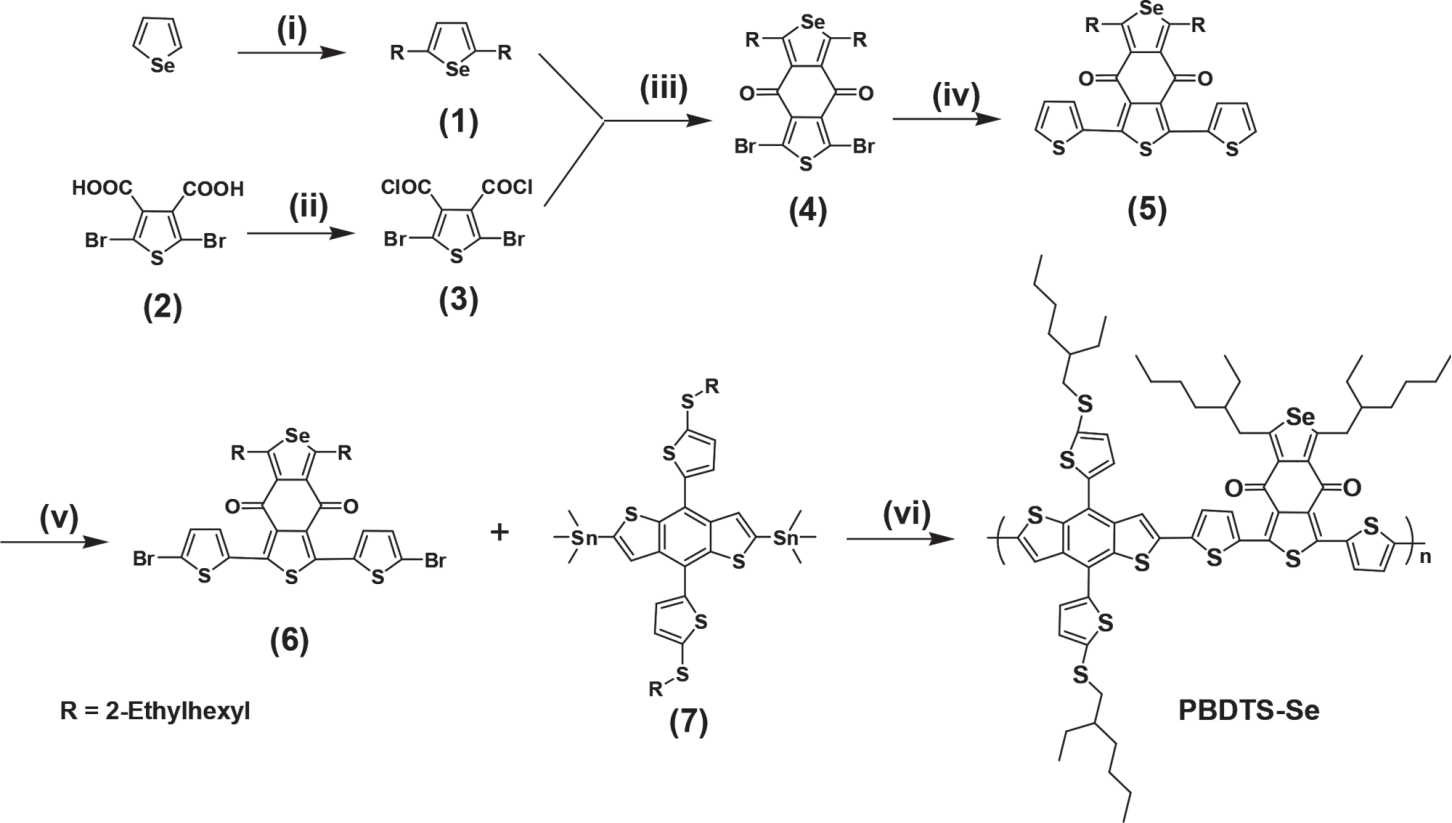
Synthetic procedure of PBDTS‐Se. Reagents and conditions: i) *n*‐butyllithium, tetrahydrofuran (THF), 2‐ethylhexyl bromide; ii) oxalyl chloride, dichloromethane; iii) aluminium trichloride, dichloromethane; iv) trimethyl(thiophen‐2‐yl)stannane, Pd(PPh_3_)_4_, toluene; v) *N*‐bromobutanimide, chloroform; and vi) Pd(PPh_3_)_4_, toluene, inert atmosphere, reflux, 8 h.

The UV–vis absorption spectra of PBDTS‐Se, SdiPBI‐S, and their blend film (1:1, w/w) are shown in Figure 1c. The two absorption peaks of PBDTS‐Se film were located at 585 and 627 nm, which are slightly redshifted from those in solution (Figure S2, Supporting Information). It should be noted that the absorption of PBDTS‐Se is in the wavelength range of 400–700 nm, which complements the absorption spectra of SdiPBI‐S, leading to a broad absorption from 300 to 700 nm. Solar cells were fabricated with a simple conventional device structure: indium tin oxide (ITO)/poly(3,4‐ethylenedioxythiop­hene):poly(styrenesulfonate) (PEDOT:PSS)/PBDTS‐Se:SdiPBI‐S/Ca/Al (Figure 1b). The energy levels of each material used in the device are shown in Figure 1d. The HOMO and LUMO levels of PBDTS‐Se film can be obtained from the electrochemical measurements and the values are 5.34 and 3.53 eV, respectively (Figure S3, Supporting Information). The LUMO level of SdiPBI‐S film is determined to be 3.92 eV. There is a large energy offset, 1.42 V, between the HOMO of PBDTS‐Se and the LUMO of SdiPBI‐S which is preferred for a high *V*_oc_. Steady‐state photoluminescence (PL) measurements were performed on the neat and BHJ films (1:1, w/w). Compared to PBDTS‐Se and SdiPBI‐S neat films, the BHJ film showed strong PL quenching up to 98% and 99% comparing to neat film PL, indicative of highly efficient charge transfer between PBDTS‐Se and SdiPBI‐S in BHJ films.

In solar cell fabrication, PBDTS‐Se:SdiPBI‐S BHJ films of different blending ratios and of different 1, 8‐diiodooctane (DIO) contents were used. The device parameters are summarized in **Table** **1** and Table S1 (Supporting Information). The current density–voltage (*J–V*) curves and the corresponding incident photon conversion efficiency (IPCE) spectra of solar cells are shown in **Figure** **2** and Figure S4 (Supporting Information), respectively. The optimal PBDTS‐Se:SdiPBI‐S weight ratio was found to be 1:1. For as cast thin films without using a DIO additive, PBDTS‐Se:SdiPBI‐S solar cells achieved an PCE of 7.59%, with a *V*_oc_ of 0.94 V, a *J*_sc_ of 12.20 mA cm^−2^, and a FF of 66.2%. The addition of DIO can further increase the cell efficiency. When processed from 0.5% DIO, solar cells exhibited the best photovoltaic performance with a *V*_oc_ of 0.91 V, a *J*_sc_ of 12.90 mA cm^−2^, and a FF of 70.0%, leading to a PCE of 8.22%. We obtained an average efficiency of 8.01% for more than 20 devices under this condition, indicating the excellent reproducibility of device fabrication. A PCE of 8.22% is among the highest values reported in the literature for non‐fullerene organic solar cells, a distinct improvement over our recent report of SdiPBI‐S‐based solar cells with a PCE of 7.16%, a *V*_oc_ of 0.90 V, a *J*_sc_ of 11.98 mA cm^−2^, and a FF of 66.1%. By comparison, the improved PCE mainly comes from a higher *J*_sc_ and a higher FF. The high *J*_sc_ is ascribed to the high conversion efficiency of the absorbed photons into electrons in the device. As seen clearly from Figure 2b, a very high IPCE value of over 60% over a wide optical range from 400 to 660 nm is obtained, showing a maximum peak over 70% at 440 nm. The calculated *J*_sc_ value (12.62 mA cm^−2^) from IPCE spectrum is in agreement with the value (12.90) measured from the *J–V* curve. The high FF of 70.0% indicates a good active layer morphology that enables efficient charge transport and charge collection in devices. The charge mobilities of PBDTS‐Se neat and BHJ films were measured using the space‐charge‐limited current (SCLC) method.^32^ The hole mobility of PBDTS‐Se and the electron mobility of SdiPBI‐S in BHJ film are 1.2 × 10^−3^ and 3.3 × 10^−3^ cm^2^ V^−1^ s^−1^, respectively (Figure S5, Supporting Information). The addition of 0.5% DIO into BHJ films increased both the hole and electron mobilities to values of 1.9 × 10^−3^ and 3.5 × 10^−3^ cm^2^ V^−1^ s^−1^, respectively, which are comparable to the mobility values of their neat films (Figure S6, Supporting Information). The high mobilities in the BHJ thin films are similar to those of the neat materials, suggesting good network formation for each component. The balanced mobility in BHJ films contributes to the high *J*_sc_ and FF in solar cells devices.

**Table 1:** Summary of device parameters of PBDTS‐Se:SdiPBI‐S solar cells with different DIO concentrations under the illumination of AM 1.5 G, 100 mW cm^−2^

DIO [v/v]	*V*_oc_ [V]	*J*_sc_ [mA cm^−2^]	FF [%]	PCE^a)^ [%]	PCE_max_ [%]
0%	0.943 ± 0.007	12.23 ± 0.16	66.2 ± 1.8	7.50 ± 0.23	7.59
0.3%	0.919 ± 0.006	12.46 ± 0.16	67.1 ± 0.9	7.69 ± 0.15	7.72
0.5%	0.909 ± 0.006	12.80 ± 0.25	68.8 ± 1.3	8.01 ± 0.20	8.22
1.0%	0.897 ± 0.008	12.50 ± 0.10	66.8 ± 0.3	7.41 ± 0.12	7.49

^a)^The average PCE value was calculated from ten devices for each condition.

**Figure 2:**
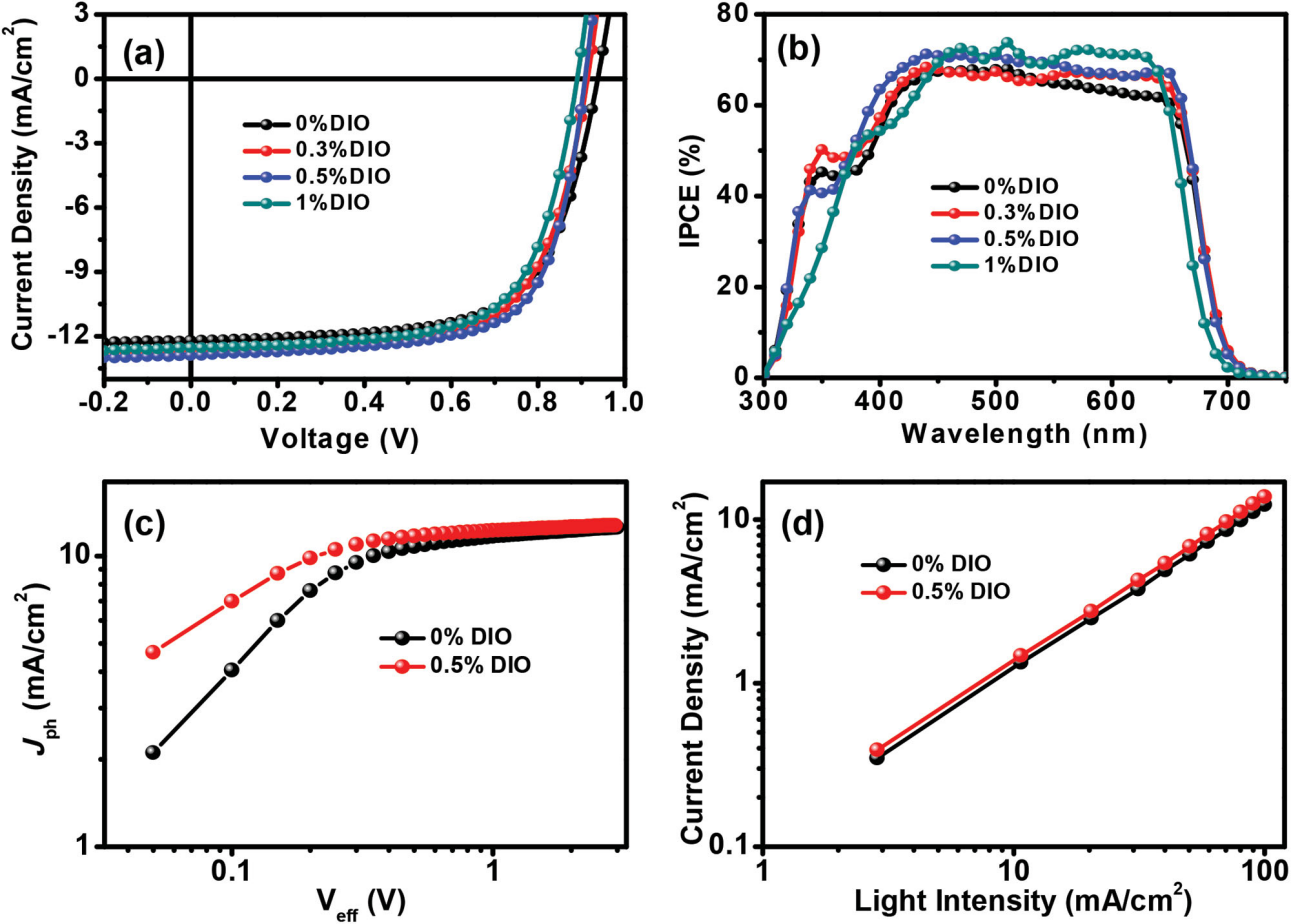
a) *J–V* curves of PBDTS‐Se:SdiPBI‐S solar cells with different DIO concentrations under simulated AM 1.5 G irradiation (100 mW cm^−2^) and b) the corresponding IPCE spectra. c) Photocurrent density (*J*_ph_) versus effective voltage (*V*_eff_) characteristics of PBDTS‐Se:SdiPBI‐S solar cells with and without 0.5% DIO. d) Short‐circuit density (*J*_sc_) versus light intensity characteristics of PBDTS‐Se:SdiPBI‐S solar cells with and without 0.5% DIO.

To study the exciton dissociation and charge collection in solar cells, the photocurrent (*J*_ph_) versus the effective applied voltage (*V*_eff_) analysis was measured. *J*_ph_ can be obtained by subtracting the dark current from the current under illumination and *V*_eff_ can be obtained by subtracting the applied voltage from the voltage where *J*_ph_ is 0.^33^ At a high *V*_eff_ value (≥2 V), *J*_ph_ is saturated, since the recombination is minimized due to the high internal electric field in the cell. The charge dissociation probability (*P*(*E,T*)) can be estimated from the value of *J*_ph_/*J*_sat_. Under the short‐circuit and maximal power output conditions, the *P*(*E,T*) values are 95%, 97%, and 78%, 84% for solar cells with and without 0.5% DIO additive, respectively. The results indicate that both cells (with and without DIO) have a high exciton dissociation rate and a more efficient charge collection. We noted that *J*_ph_ showed a linear dependence on light intensity with the slope equal to 1, indicating a very weak bimolecular recombination in the devices.^34^

It is known that the performance of solar cells strongly depends on the morphology of the active layer. We characterized the structure order of PBDTS‐Se and SdiPBI‐S in neat and BHJ films using grazing incidence X‐ray diffraction (GIXD).^35^, ^36^ The 2D diffraction images are shown in **Figure** **3**a–d, from which the crystalline ordering and crystal orientation in thin films can be accessed. As shown in Figure 3a (neat PBDTS‐Se film), a high intensity diffraction peak is seen in the in‐plane direction, at 0.26 Å^−1^, corresponding to a distance of 2.41 nm. This peak comes from the (100) packing of the alkyl chain in PBDTS‐Se. A strong π–π stacking peak was observed in the out‐of‐plane direction, located at 1.72 Å^−1^, giving a π–π stacking of 0.36 nm. Thus, PBDTS‐Se assumes a “face‐on” orientation. The (100) crystal size is calculated to be 9.98 nm (4.1 stacks) and the π–π stacking crystal size is calculated to be 2.10 nm (5.8 stacks). SdiPBI‐S molecule features two PBI aromatic rings tethered by a single bond and, thus, has a twisted molecular geometry, which induces steric hindrance that retards π–π stacking. In the GIXD profiles, a (100) diffraction at 0.35 A^−1^ is seen, corresponding to a distance of 1.77 nm. This distance correlates well with the hexyl substituent side chains interspacing on PBI moieties. The azimuthal distribution of the (100) peak is uniform, indicating that the SdiPBI‐S crystallites are, on average, randomly orientated in thin film. It is observed that PBI‐based crystalline solids usually showed a close π–π stacking distance due to the large aromatic planes. However, in the current case, no obvious diffraction peak is observed in diffraction image. Only a diffusive ring is seen at ≈1.4 A^−1^, corresponding to a distance of 4.83 nm. This distance is larger than usual for a π–π stacking and, thus, charge transport relies on charge hopping from one PBI molecule into the adjacent one through head‐to‐center contact. In this light, the transport can be isotropic. When blending PBDTS‐Se with SdiPBI‐S, crystalline features from both materials are seen. For BHJ film without DIO, the in‐plane line cut profile in low *q* region showed a broad peak that is a sum of the diffraction from PBDTS‐Se and SdiPB1‐S. π–π stacking from PBDTS‐Se is seen in the out‐of‐plane direction, and thus the face‐on orientation is preserved in the film of the blend. In the in‐plane direction, two reflections located at 0.27 and 0.32 A^−1^ are seen in the low *q* region, which arise from the (100) planes of PBDTS‐Se and SdiPBI‐S. The crystal size for PBDTS‐Se, determined from the width of the (100) peak, is 10.44 nm, and for SdiPBI‐S it is 4.04 nm. The π–π stacking reflection at 1.72 A^−1^ yielded a crystal size of 2.22 nm. The crystal size for SdiPBI‐S is significantly smaller in comparison to PBDTS‐Se polymer counterparts. It should be noted that the packing distance for these materials are slightly different, which is probably due to the interactions of the donor and acceptor during casting. When DIO additive was used, the crystalline behavior was not significantly changed and similar diffraction profiles are seen. The position of the (100) peak of PBDTS‐Se remained constant but the width decreased yielding a larger crystal size of 12.85 nm. The (100) peak of SdiPBI‐S peak shifted to 0.31 A^−1^ with a similar crystal size. However, the π–π stacking PBDTS‐Se under DIO processing shifted to 1.74 A^−1^, corresponding to a distance of 0.36 nm. Its crystal size also increased to 2.60 nm. Thus, it is evident that DIO improved the interchain packing of PBDTS‐Se along both the (100) and (001) (π–π stacking) directions, giving rise to an improved charge transport and, consequently, improved carrier transport and collection efficiency. The results agree well with the SCLC measurements.

**Figure 3:**
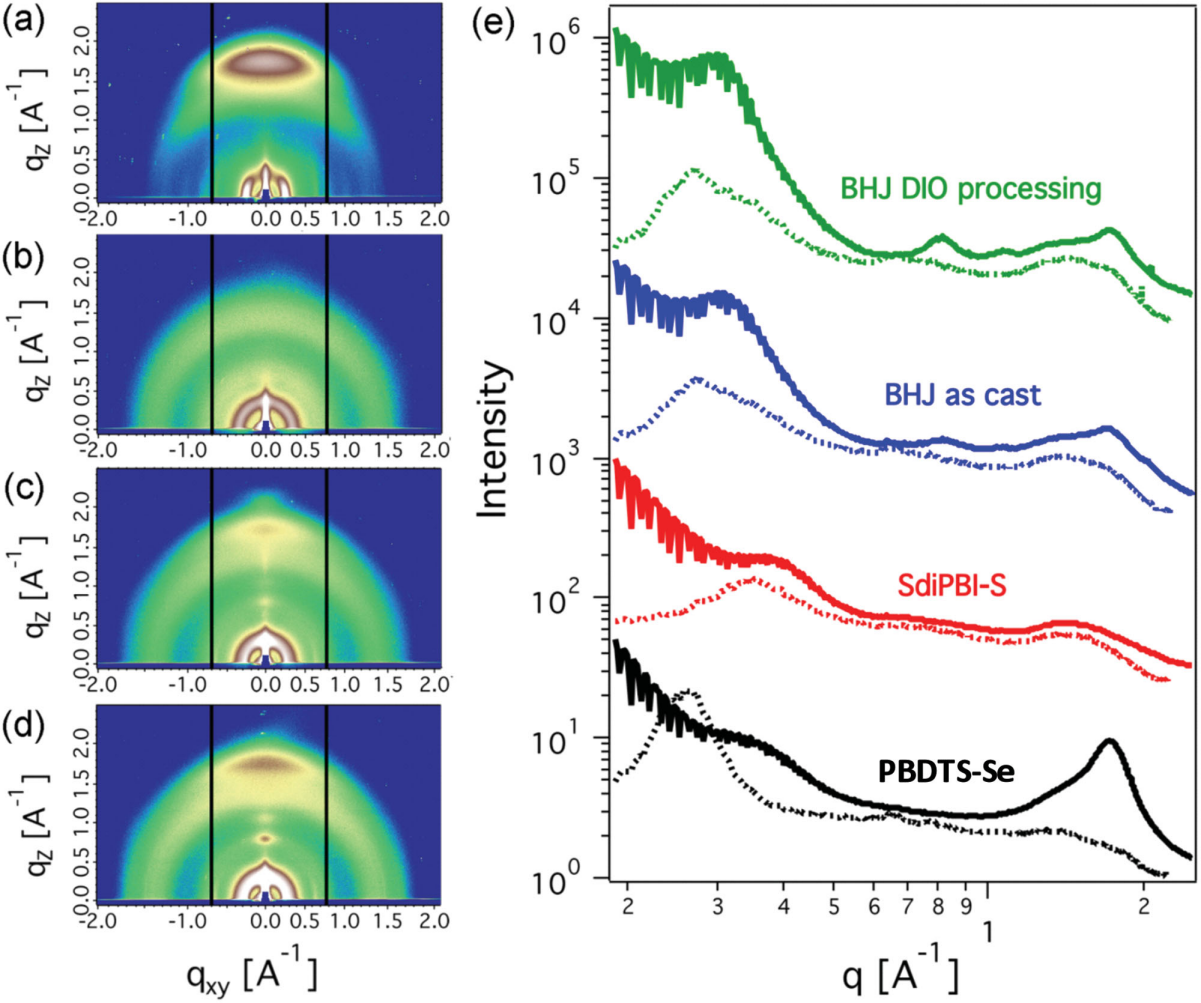
Grazing incidence X‐ray diffraction of neat and BHJ films. a) PBDTS‐Se. b) SdiPBI‐S. c) BHJ film without DIO. d) BHJ film with 0.5% DIO. e) Line‐cut profiles of GIXD results. Solid line: out‐of‐plane line cut; dotted line: in‐plane line cut.

The feature of phase separation of this BHJ blends was studied by using resonant soft X‐ray scattering (RSoXS).^37^ The near edge X‐ray absorption fine structure spectra for PBDTS‐Se and SdiPBI‐S are shown in Figure S8 (Supporting Information). Similar spectra were seen for PBDTS‐Se and SdiPBI‐S, since both polymers have similar atomic compositions. Figure S9 (Supporting Information) shows the energy scans around the carbon K‐edge and **Figure** **4** summarizes the scattering profiles at a photon energy of 286.8 eV where the highest contrast was observed. For BHJ film without DIO additive, a slowly decaying scattering profiles is seen, which suggests that a crystalline network structure has not formed. In contrast, when 0.5% DIO is added, the scattering profile showed a broad maximum from 0.01 to 0.02 A^−1^, corresponding to a length scale of 30–60 nm. And thus a network work structure is formed in the BHJ thin film which is also reflected in the atomic force microscopy (AFM) results. As shown in Figure S7 (Supporting Information), the surface of the PBDTS‐Se is dominated by a fibrillar texture. This feature is preserved in the solution cast BHJ blends. When DIO is used, the amplitude of the surface features increased and fibrillar texture is more pronounced. These results correlate well with the GIXD and RSoXS results, indicating that the DIO enhanced crystallization and phase separation, which led to the high PCE of 8.2%.

**Figure 4:**
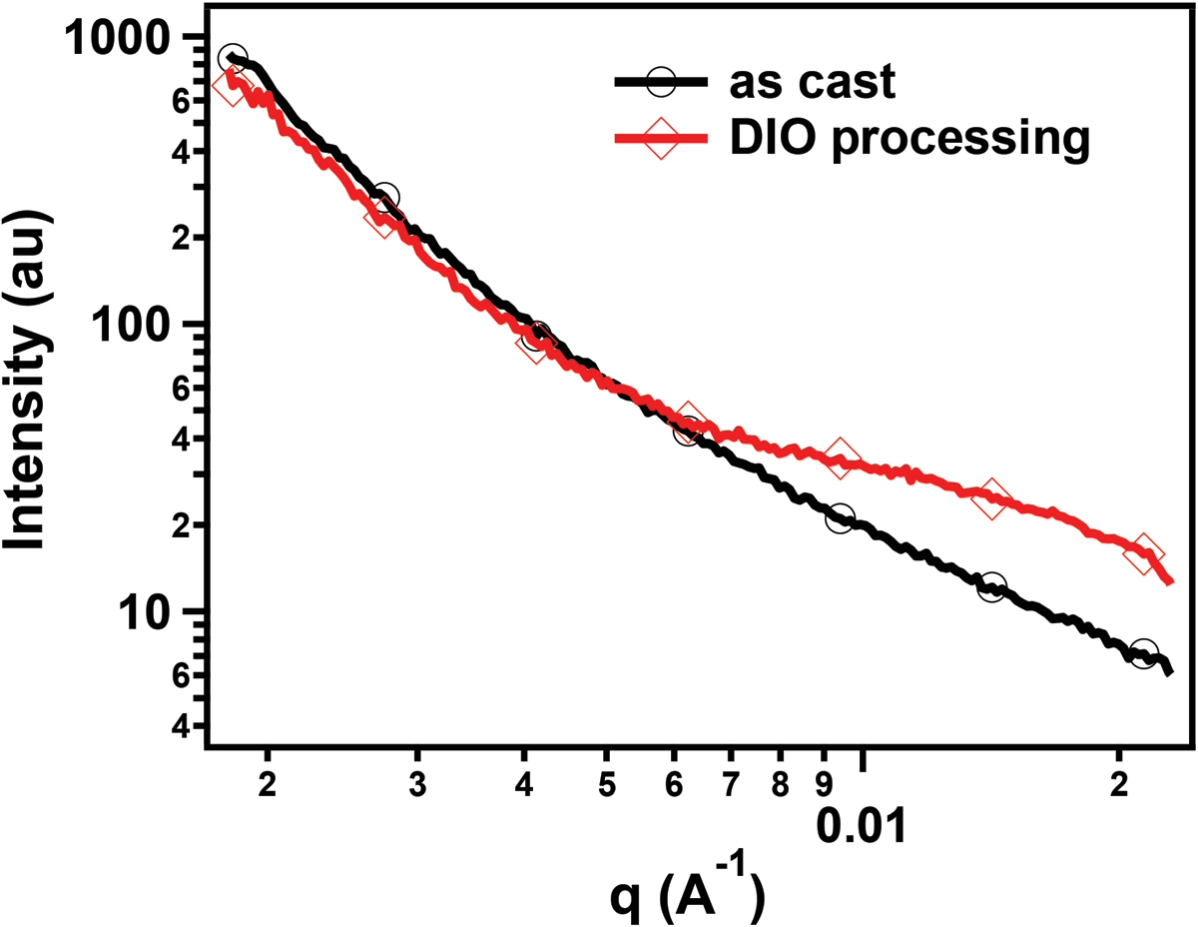
Resonant soft X‐ray scattering of PBDTS‐Se:SdiPBI‐S blend films with and without 0.5% DIO.

In summary, we demonstrated a novel polymer donor named PBDTS‐Se. Through the rational molecular design, PBDTS‐Se has a broad absorption (from 300 to 700 nm), a high charge carrier mobility of 2.6 × 10^−3^ cm^2^ V^−1^ s^−1^, and a relatively low HOMO level of 5.34 eV. When combined with the SdiPBI‐S acceptor, solar cells have a *V*_oc_ of 0.91 V, a high *J*_sc_ of 12.90 mA cm^−2^, and a high FF of 70.0%, leading to a PCE of 8.22%, which is among the highest reported values. The broad absorption, high and balanced hole/electron mobility, and good morphologies with small phase‐separated domain sizes in the BHJ films indicate that PBDTS‐Se donor matches well with SdiPBI‐S acceptor, in terms of optical, electronic, and morphological properties. Our results show that even for a give non‐fullerene acceptor, rational design of novel donor materials can lead to further improve its photovoltaic performance.

## Experimental Section

*Solar Cell Fabrication and Characterization*: ITO substrates were cleaned subsequently with detergent, deionized water, acetone, and isopropyl alcohol. After 10 min of UV ozone treatment, PEDOT:PSS (Heraeus Clevios P VP A 4083) was spin casted onto the ITO substrate at 4000 rpm for 40 s and then dried at 150 °C for 10 min in air. The thickness of PEDOT:PSS is about 40 nm, determined with a Dektak XT stylus profilometer. PBDTS‐Se was codissolved with SdiPBI‐S in 1,2‐dichlorobenzene (DCB) with different weight ratio and DIO contents (the concentration of PBDTS‐Se is fixed at 9 mg mL^−1^ for all mixed solutions). The active layer was formed by spin coating the mixed solution atop PEDOT:PSS layer at 1000 rpm for 90 s. After that, the samples were annealed at 100 °C for 5 min on a hot plate in a glove box. The optimal thickness of the champion cell is about 120 nm, measured on a Dektak XT stylus profilometer. Finally, a 10 nm thick Ca layer and a 100 nm thick Al were sequentially deposited by thermal evaporation through a shadow mask at a vacuum of 5 × 10^−5^ Pa. The active area of the devices was 4.50 mm^2^. During the measurement, an aperture with the area of 3.14 mm^2^ was used. Current density–voltage (*J–V*) characteristics were measured in a Keithley 2400 Source Measure Unit. Short‐circuit current was measured in an air mass 1.5 global (AM 1.5 G) solar simulator (Class AAA solar simulator, Model 94063A, Oriel) with an irradiation intensity of 100 mW cm^−2^, which was measured by a calibrated silicon solar cell and a readout meter (Model 91150V, Newport). IPCE spectra were measured by using a QEX10 Solar Cell IPCE measurement system (PV measurements, Inc.).

*SCLC Measurements*: Charge carrier mobilities of the neat and BHJ films were measured by using space charge limit current (SCLC) method. The device structures of the hole‐ and electron‐only devices are ITO/MoO*_x_*/BHJ (or neat film)/MoO*_x_*/Al and ITO/Al/BHJ (or neat film)/Al, respectively. The mobility was determined by fitting the dark current to the model of a single carrier SCLC using the equation: *J* = 9*ε*_0_ *ε*_r_ *μV^2^/*8*d^3^*, where *J* is the current density, *d* is the film thickness of the active layer, *μ* is the charge carrier mobility, *ε*_r_ is the relative dielectric constant of the transport medium, and *ε*_0_ is the permittivity of free space. *V* = *V*_app_ – *V*_bi_, where *V*_app_ is the applied voltage and *V*_bi_ is the offset voltage. The carrier mobility can be calculated from the slope of the *J* ^1/2^ – *V* curves.

*GIXD Characterization*: It was performed at beamline 7.3.3, advanced light source (ALS), Lawrence Berkeley National Lab (LBNL). X‐ray energy was 10 keV and the scattering intensity was recorded on a 2D image plate (Pilatus 2M) with a pixel size of 172 m (1475 × 1679 pixels). The samples‐to‐detector distance was about 300 mm. The incidence angle was chosen to be 0.16°, which was above the critical angle of BHJ thin film but below critical angle of wafer substrate. The samples were prepared on PEDOT:PSS covered Si wafers in a similar manner to the devices. Resonant soft X‐ray scattering experiments were carried out in beamline 11.0.1.2, ALS, LBNL. Experiments were done in transmission geometry. BHJ thin films were flowed in water and transferred onto Silicon Nitride windows fro Norcada Inc. Samples were loaded into a high vacuum chamber (≈10^−7^ torr) to avoid carbon contaminations in ambient environment. A series of phonton energy was used in running the experiments and 300 nm polysteren spheres were used as standard to calibrate the beam center and the sample‐to‐detecter distance.

*Instrumentation*: AFM images were taken on a Dimension Icon AFM (Bruker) using a tapping mode. UV–vis absorption spectra were measured using a UV–vis spectrophotometer (Shimadzu UV‐2700). Photoluminescence spectra were measured using a Shimadzu RF‐5301PC spectrofluorophotometer.

## Supporting information

As a service to our authors and readers, this journal provides supporting information supplied by the authors. Such materials are peer reviewed and may be re‐organized for online delivery, but are not copy‐edited or typeset. Technical support issues arising from supporting information (other than missing files) should be addressed to the authors.

Supplementary
