# Supplementary material for: High‐Performance Non‐Fullerene Organic Solar Cells Based on a Selenium‐Containing Polymer Donor and a Twisted Perylene Bisimide Acceptor
Source: Adv Sci (Weinh). 2016 Apr 23;3(9):1600117. doi: 10.1002/advs.201600117 (PMC5039968; doi:10.1002/advs.201600117)
Supplement: Supplementary file 1 — Supplementary [file ADVS-3-0l-s001.pdf]

Copyright WILEY-VCH Verlag GmbH & Co. KGaA, 69469 Weinheim, Germany, 2016.

Supporting Information

for *Adv. Sci.,* DOI: 10.1002/advs.201600117

High-Performance Non-Fullerene Organic Solar Cells Based on a Selenium-Containing Polymer Donor and a Twisted Perylene Bisimide Acceptor

Tao Liu, Dong Meng, Yunhao Cai, Xiaobo Sun, Yan Li, Lijun Huo, Feng Liu,* Zhaohui Wang, Thomas P. Russell, and Yanming Sun**

Copyright WILEY-VCH Verlag GmbH & Co. KGaA, 69469 Weinheim, Germany, 2013.

Supporting Information

High Performance Non-Fullerene Organic Solar Cells Based on a Selenium-Containing Polymer Donor and a Twisted Perylene Bisimide Acceptor

Tao Liu, Dong Meng, Yunhao Cai, Xiaobo Sun, Yan Li, Lijun Huo, Feng Liu, * Zhaohui Wang, Thomas P. Russell, and Yanming Sun**

T. Liu, Prof. L. Huo, Y. Cai, Prof. X. Sun, Prof. Y. Sun
Heeger Beijing Research and Development Center
School of Chemistry and Environment
Beihang University
Beijing 100191, PR China.
E-mail:huolijun@iccas.ac.cn; sunym@buaa.edu.cn

Dr. F. Liu
Materials Science Division
Lawrence Berkeley National Lab
Berkeley 94720, USA
E-mail:iamfengliu@gmail.com

D. Meng, Dr. Y. Li, Prof. Z. Wang
Beijing National Laboratory for Molecular Science
Key Laboratory of Organic Solids
Institute of Chemistry, Chinese Academy of Sciences
Beijing 100190, P. R. China

Prof. T. P. Russell
Polymer Science and Engineering Department
University of Massachusetts
Amherst, MA 01003 , USA

*Synthesis of 2,5-di(octan-3-yl)selenophene (**1**):* In a 250 mL argon purged flask, n-butyl lithium hextane solution (2.5M, 53.1 mL) was added into a solution of selenophene (5.8 g, 44 mmol) in THF (50 mL) at 0 °C and the mixture was warmed up to 50 °C and stirred for 2 hour. After cooling down to room temperature, 3-(bromomethyl)heptane (29.7 g, 154 mmol) was injected one potion and the mixture was stirred overnight at the 50 °C and then poured into ice water. The mixture was extracted by ethyl ether twice and the combined organic phase was concentrated to obtain the raw compound 1. Further purification was carried out by distill to obtain pure compound 1 as liquid (12.7 g, yield 81%). ^{1}H NMR ($CDCl_3$, 400MHz), δ (ppm): δ 6.71 (s, 2H), 2.78 (d, 4H), 1.55 (s, 2H), 1.46-1.33 (br, 16H), 0.93-0.90 (br, 12H). ^{13}C NMR ($CDCl_3$, 100MHz), δ (ppm): 149.2, 126.6, 42.3, 37.1, 32.6, 29.1, 25.7, 23.3, 14.2, 11.0. Anal. Caled for $C_{20}H_{36}Se$ (%): C, 67.58; H, 10.21; Found: C, 65.68; H, 9.85. MS: m/z Caled for 356.0; Found 356.0.

*Synthesis of 1,3-dibromo-5,7-bis(2-ethylhexyl)-4H,8H-selenopheno[3',4':4,5]benzo[1,2-c]thiophene-4,8-dione (**4**):* In a 100 mL argon purged flask, oxalyl chloride (10 mL) and one drop of N,N-Dimethylformamide (DMF) was injected into compound 2[1] (2 g, 6.06 mmol) in toluene (30 mL) at room temperature and the mixture was warmed up to 65 °C and stirred for 2 hour. The mixture was concentrated to obtain the raw compound 3.[1] In a 100 mL flask, aluminium trichloride (3.57 g, 26.67 mmol) was slowly added into a solution of raw compound 3 and compound 1 (2.15 g, 6.06 mmol) in anhydrous dichloromethane (50 mL) at 0 °C and stirred for 30 minutes. Then the mixture was poured into ice water and extracted by dichloromethane twice and the combined organic phase was concentrated to obtain the raw compound 4. Further purification was carried out by a silica gel column using dichloromethane/ petroleum ether (1/6) eluent to obtain pure compound 4 as pale yellow solid (0.7 g, yield 18%). ^{1}H NMR ($CDCl_3$, 400MHz), δ (ppm): δ 3.45 (s, 4H), 1.67 (s, 2H), 1.38-1.30 (br, 16H), 1.13-0.76 (br, 12H). ^{13}C NMR ($CDCl_3$, 100MHz), δ (ppm):

176.2, 166.1, 135.2, 134.7, 119.3, 43.0, 36.9, 33.0, 29.0, 26.3, 23.1, 14.2, 11.1. Anal. Caled for $C_{26}H_{34}Br_2O_2SSe$ (%): C, 48.09; H, 5.28; Found: C, 48.06; H, 5.70. MALDI-TOF MS: m/z Caled for 649.9; Found 649.0.

*Synthesis of 5,7-bis(2-ethylhexyl)-1,3-di(thiophen-2-yl)-4H,8H-selenopheno[3',4':4,5]benzo[1,2-c]thiophene-4,8-dione (**5**):* In a 50 mL argon purged flask, compound 4 (0.5 g, 0.77 mmol) was added into a solution of trimethyl(thiophen-2-yl)stannane (0.86 g, 2.31 mmol) in toluene (20 mL) at room temperature. When the flask was purged by argon for 5 minutes, 90 mg of $Pd(PPh_3)_4$ was added as catalyst, and then the mixture was purged by argon for another 15 minutes. The reactant was heated to reflux overnight. Then the mixture was concentrated to obtain the raw compound 5. Further purification was carried out by a silica gel column using dichloromethane/ petroleum ether (1/6) eluent to obtain pure compound 5 as orange solid (0.4 g, yield 79%). ^{1}H NMR ($CDCl_3$, 400MHz), δ (ppm): δ 7.72 (d, 2H), 7.48 (d, 2H), 7.11 (d, 2H), 3.44 (t, 4H), 1.65 (m, 2H), 1.45-1.26 (br, 16H), 0.95-0.89 (br, 12H).^{13}C NMR ($CDCl_3$, 100MHz), δ (ppm): 178.5, 164.1, 142.2, 135.6, 133.7, 132.7, 130.5, 129.3, 127.3, 43.0, 36.6, 33.1, 29.1, 26.3, 23.2, 14.3, 11.1. Anal. Caled for $C_{34}H_{40}O_2S_3Se$ (%): C, 62.27; H, 6.15; Found: C, 64.22; H, 6.57. MALDI-TOF MS: m/z Caled for 656.1; Found 657.1.

*Synthesis of 1,3-bis(5-bromothiophen-2-yl)-5,7-bis(2-ethylhexyl)-4H,8H-selenopheno[3',4':4,5]benzo[1,2-c]thiophene-4,8-dione(**6**):* In a 50 mL flask, N-bromosuccinimide (NBS) (0.54 g, 3.05 mmol) was slowly added into a solution of compound 5 (1 g, 1.53 mmol) in chloroform (10 mL) at room temperature and stirred for 2 hours and then poured into ice water. The mixture was extracted by dichloromethane twice and the combined organic phase was concentrated to obtain the raw compound Se-1. Further purification was carried out by a silica gel column using dichloromethane/ petroleum ether (1/10) eluent to obtain pure compound 6 as orange solid (0.94 g, yield 75.6%).^{1}H NMR ($CDCl_3$, 400MHz), δ (ppm): δ 7.39 (d, 2H),

7.03 (d, 2H), 3.42 (m, 4H), 1.68 (d, 2H), 1.45-1.26 (br, 16H), 0.96-0.89 (br, 12H). ^{13}C NMR ($CDCl_3$, 100MHz), δ (ppm): 178.2, 164.7, 141.3, 135.1, 134.9, 132.0, 130.3, 129.8, 118.2, 42.9, 36.7, 33.2, 29.2, 26.4, 23.2, 14.3, 11.1. Anal. Caled for $C_{34}H_{38}Br_2O_2S_3Se$ (%): C, 50.19; H, 4.71; Found: C, 50.38; H, 4.81. MALDI-TOF MS: m/z Caled for 813.9; Found 813.0.

Synthesis of PBDTS-Se: The compound 6 (0.14 mmol) and the monomer of 7[2] (0.14 mmol) were mixed in 10 ml of toluene. When the flask was purged by argon for 5 minutes, 15 mg of $Pd(PPh_3)_4$ was added as catalyst, and then the mixture was purged by argon for another 5 minutes. The reactant was heated to reflux for 8 hours. When the reactant was cooled to room temperature, the polymer was precipitated by addition of 100 ml methanol, and then filtered through a Soxhlet thimble, which was then subjected to Soxhlet extraction with methanol, hexane, and chloroform. The corresponding polymer was recovered as solid powder from the chloroform fraction by precipitation from methanol. After the polymers were dried under vacuum, the yields and molecular weight are as follows: PBDTS-T2: Yield: 53%. ^1H NMR ($CDCl_3$, 400MHz), δ (ppm): δ 7.89-6.71 (br, 10H), 3.75-2.71 (br, 8H), 1.78-0.67 (br, 60H). Anal. Caled for $C_{68}H_{78}O_2S_9Se$ (%): C, 63.08; H, 6.07; Found: C, 63.11; H, 6.04. Weight average molecular weight (M_w) and polydispersity index (PDI) estimated from GPC are 33.5 kDa and 1.82, respectively.

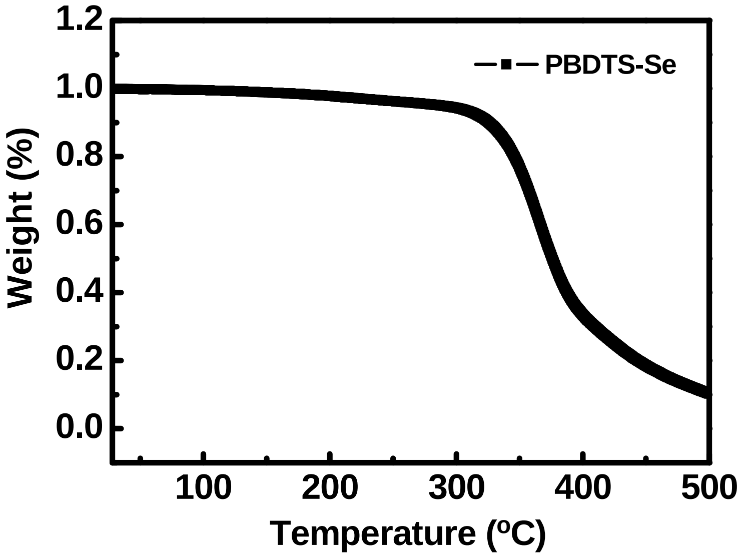

Figure S1. Thermogravimetric (TGA) plot of PBDTS-Se with a heating rate of 10 °C/min under the inert atmosphere.

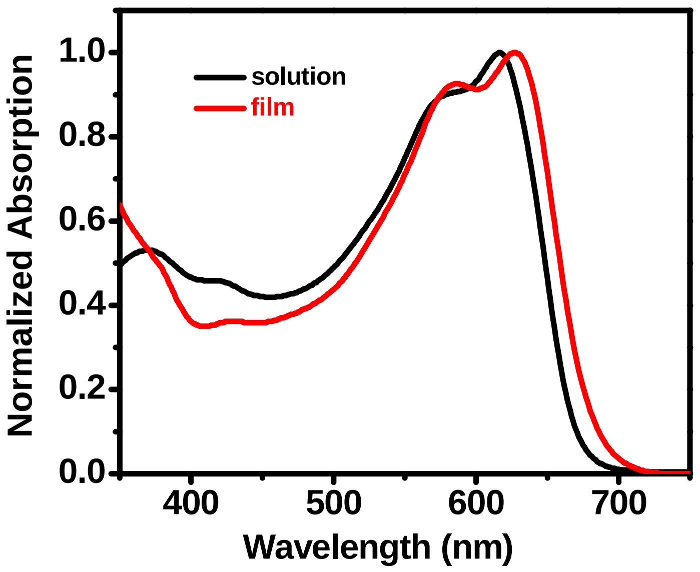

Figure S2. UV-vis absorption spectra of PBDTS-Se in solution and thin film.

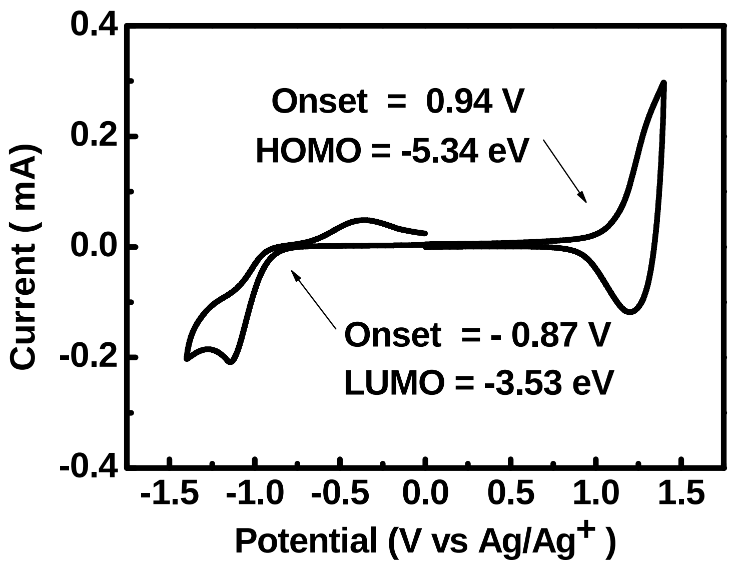

Figure S3. The electrochemical cyclic voltammetry measurement of PBDTS-Se. The HOMO and LUMO levels calculated from the onset oxidation and reduction potentials are -5.34 eV and -3.53 eV, respectively.

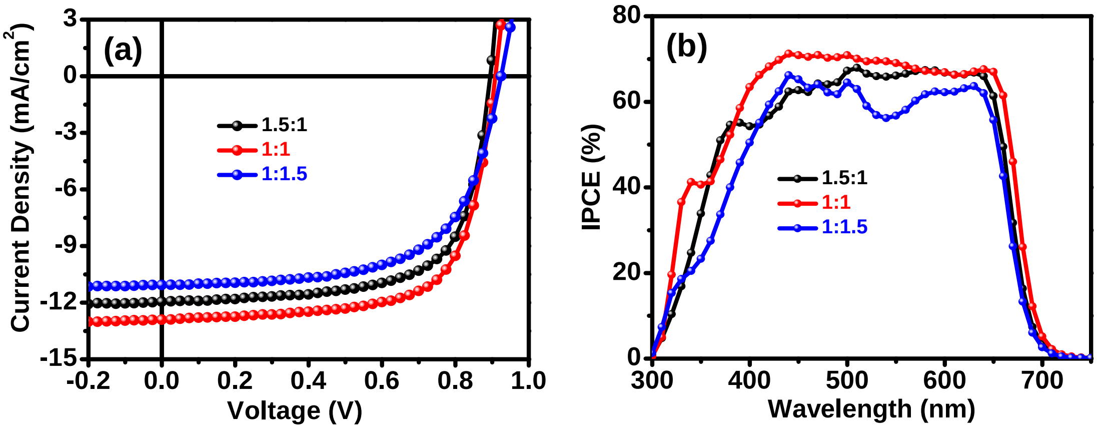

Figure S4. a) *J–V* curves of PBDTS-Se:SdiPBI-S solar cells with different weight ratios (0.5% DIO) under the illumination of AM1.5G, 100 mW/cm^2 and b) the corresponding IPCE spectra.

Table S1. Summarized device parameters of PBDTS-Se:SdiPBI-S solar cells with different blend ratios under the illumination of AM1.5G, 100 mW/cm^2.

DIO (v/v)	V_{oc} (V)	J_{sc} (mA/cm^2)	FF (%)	PCE (%)[a]	PCE_{max} (%)
1.5:1	0.902±0.007	11.73±0.22	67.9±0.6	7.18±0.11	7.28
1:1	0.909±0.006	12.80±0.25	68.8±1.3	8.01±0.20	8.22
1:1.5	0.921±0.004	10.96±0.10	63.7±0.6	6.43±0.10	6.46

[a]The average PCE value was calculated from 10 devices for each condition.

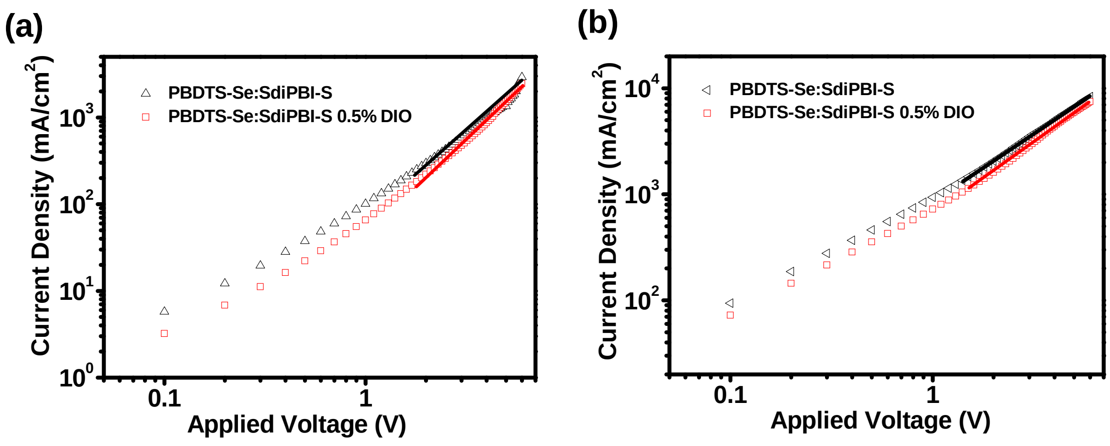

Figure S5. The experimental current density-applied voltage (*J-V*) characteristics for a) hole-only device, and b) electron-only device for PBDTS-Se:SdiPBI-S blend films with and without 0.5% DIO.

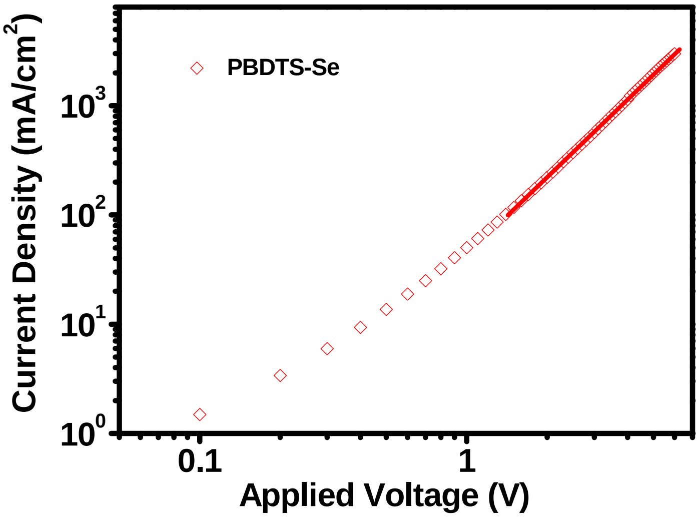

Figure S6. The experimental current density-applied voltage (*J*-*V*) characteristics for PBDTS-Se film. The hole mobility was calculated to be 2.6×10^{-3} cm^2 V^{-1} s^{-1}.

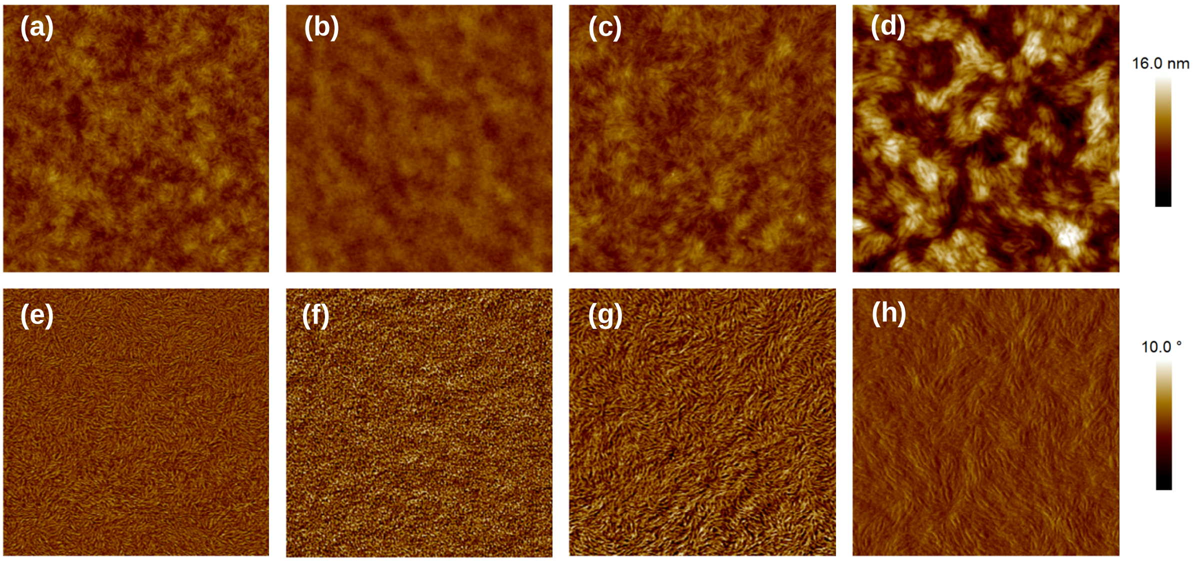

Figure S7. AFM height and phase images (2 × 2 μm) of (a, e) PBDTS-Se neat film, (b, f) SdiPBI-S neat film, (c, g) PBDTS-Se:SdiPBI-S blend films (1:1, w/w) without DIO and (d, h) PBDTS-Se:SdiPBI-S blend films (1:1, w/w) with 0.5% DIO.

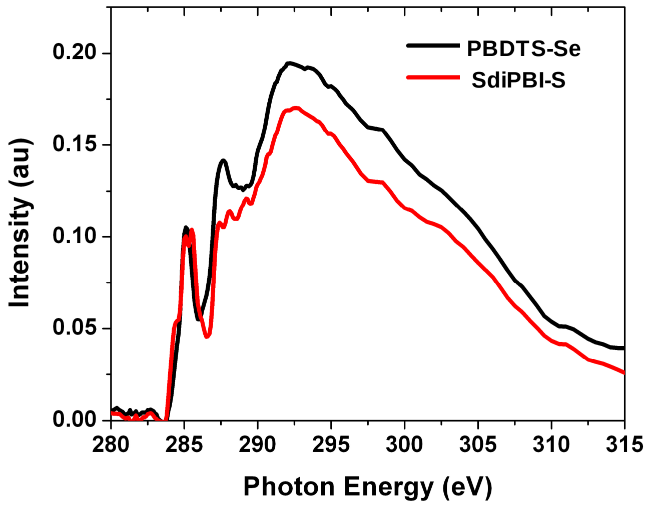

Figrue S8. NEXAFS of PBDTS-Se and SdiPBI-S.

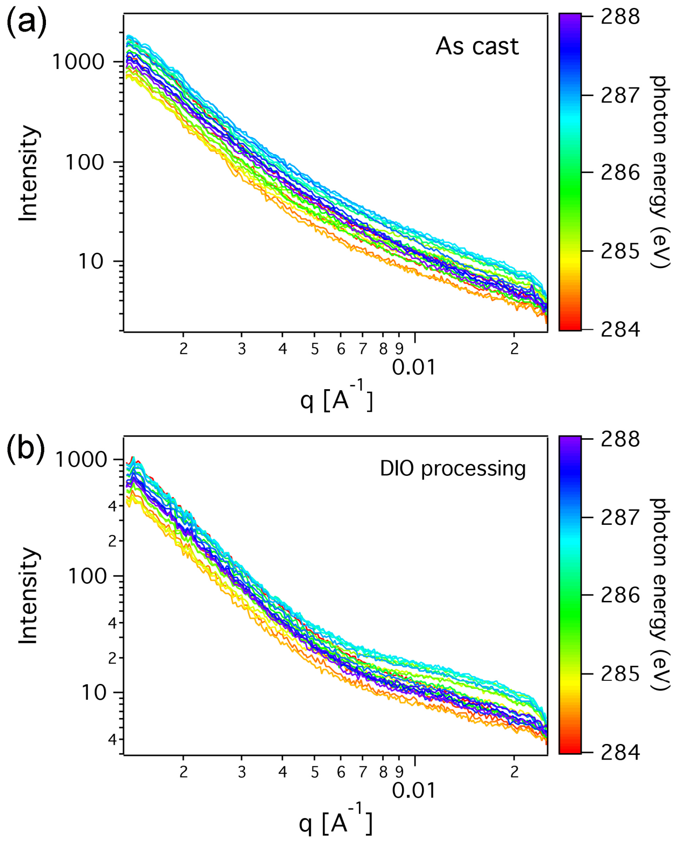

Figrue S9. RSoXS of PBDTS-Se:SdiPBI-S blends using different photon energies: (a) as cast film; (b) DIO processed film.

References:

[1] Y. Ie, J. Huang, Y. Uetani, M. Karakawa, Y. Aso, *Macromolecules* **2012**, *45*, 4564.

[2] C. Cui, W.-Y. Wong, Y. Li, *Energy Environ. Sci.* **2014**, *7*, 2276.
